# Macrophages and Uveitis in Experimental Animal Models

**DOI:** 10.1155/2015/671417

**Published:** 2015-05-20

**Authors:** Salvador Mérida, Elena Palacios, Amparo Navea, Francisco Bosch-Morell

**Affiliations:** ^1^Instituto de Ciencias Biomédicas, Universidad CEU Cardenal Herrera, 46113 Valencia, Spain; ^2^FISABIO, Oftalmología Médica, 46020 Valencia, Spain

## Abstract

Resident and infiltrated macrophages play relevant roles in uveitis as effectors of innate immunity and inductors of acquired immunity. They are major effectors of tissue damage in uveitis and are also considered to be potent antigen-presenting cells. In the last few years, experimental animal models of uveitis have enabled us to enhance our understanding of the leading role of macrophages in eye inflammation processes, including macrophage polarization in experimental autoimmune uveoretinitis and the major role of Toll-like receptor 4 in endotoxin-induced uveitis. This improved knowledge should guide advantageous iterative research to establish mechanisms and possible therapeutic targets for human uveitis resolution.

## 1. Introduction

The eye, like the brain, implanted uterus, and testis, is one of the body's immune privilege sites. Thus, it presents evolutionary adaptation designed to protect itself from blinding influences of immunologic inflammation. Specifically, intraocular structures and the cornea have immune privilege, but the extraocular organs and tissues that are also commonly referred to as “the eye” do not. However, this immune privilege is not always sufficient; if its natural protective mechanism fails or is overwhelmed, the eye becomes susceptible to uveitis, an intraocular inflammatory disorder that involves a wide variety of underlying etiologies.

Uveitis is the swelling and irritation of the uvea, the pigmented layer of the eye that lies beneath the sclera and cornea which comprises the iris, choroid, and ciliary body. Uveitis may also affect adjacent ocular structures, such as the retina, vitreous, and optic nerve. Its name derives from the Latin word* uva*, meaning “grape”; a peeled blue grape has a bluish vein structure that resembles the middle, vascular layer of the eye, this being the uvea [1]. Anteriorly, the iris controls pupil diameter and size and, therefore, the amount of light that reaches the retina. The ciliary body, a circular band of muscle connected to and seated immediately behind the iris, produces aqueous humor and controls lens shape. Specifically, the ciliary muscle, the circular muscle in the ciliary body, controls accommodation by relaxing or contracting zonules to enable the lens to adjust shape in order to focus. Simultaneously, it also regulates the flow of aqueous humor into Schlemm's canal. Lastly, the choroid is a highly vascular coat that extends from the optic nerve to the ora serrata and contains melanocytes, fibroblasts, macrophages, mast cells, and plasma cells.

Uveitis may be classified anatomically, pathologically, and clinically. Anatomically speaking, the current International Uveitis Study Group classifies it according to the primary anatomical location of inflammation [2, 3]: anterior uveitis (anterior chamber), intermediate uveitis (vitreous), and posterior uveitis (choroidea and retina); panuveitis (anterior chamber, vitreous, choroidea, and retina). Uveitis may also be classified according to principal pathologic features: granulomatous uveitis, characterized by blurred vision, mild pain, eye tearing, and mild sensitivity to light; nongranulomatous uveitis, with acute onset, pain, and intense sensitivity to light and a better recovery rate than granulomatous uveitis. In clinical terms, uveitis has been classified by the International Uveitis Study Group as infectious (bacteria, viral, fungal, parasitic, etc.), noninfectious (known or unknown systemic association), and masquerade (group of eye diseases that mimic chronic intraocular inflammation) [4].

In developed countries, uveitis affects about 200 per 100 000 persons in the general population. The average annual incidence of uveitis has been reported as nearly 20 per 100 000 and peaks for the 20–50-year-old age group [5]. The total population prevalence of uveitis varies geographically: 38 per 100 000 in France [6], 68–76.6 per 100 000 in Finland [6], and around 120 per 100 000 in the United States [7]. It is estimated to be 470 per 100 000 in India [8] and 620 per 100 000 in Taiwan [9]. More than half of all patients with uveitis develop complications that are related to the disease, and up to 35% of patients suffer severe visual impairment. It can cause devastating visual loss, is the fifth commonest cause of visual loss in the developed world, and accounts for about 10–15% of cases of total blindness and up to 20% of legal blindness [10, 11].

During inflammation processes and in the eye's innate immune response, monocytes and macrophages are critical regulators and effectors that work as the immediate and first arm of the immune system.

## 2. Monocytes, Macrophages, and Dendritic Cells in the Eye

In 1972, van Furth et al. [12] classified all highly phagocytic mononuclear cells and their precursors as what they labeled the “mononuclear phagocytic system.” They designated tissue macrophages, circulating monocytes, promonocytes, and their precursor cells in bone marrow as mononuclear phagocytes. Therefore, the mononuclear phagocytic system is generated from the hematopoietic stem cells located in bone marrow. Monocytes are a subset of circulating leukocytes that are released into circulation which seed tissues throughout the body within a few days. They also reach the spleen, which serves as a storage reservoir for immature monocytes. Monocytes can further differentiate into a collection of tissue macrophages and dendritic cells (DCs), probably according to the inflammatory milieu and pathogen-associated pattern recognition receptors. Thus, when monocytes leave blood vessels and extravasate through the endothelium, they differentiate into macrophages or DCs. Some usual settings of tissue macrophages include skin, connective tissue, perivascular connective tissue, and lymph nodes.

In the eye, the retina presents microglia, populations of myeloid-derived cells, principally macrophages, which are anatomically situated within the glial limitans of the inner retina vessels and throughout the parenchyma of the retina [13, 14]. So microglia express various macrophage-associated markers like CD14, CD11b, and EGF-like module-containing mucin-like hormone receptor-like 1 (EMR1; also known as F4/80 in mice) [15]. Retinal macrophages are constantly replaced. There is still some controversy as to whether the resident microglia in the retina are replaced by* in situ* proliferation and/or recruitment of myeloid cells from the bloodstream. Thus, some groups using EGFP-transgenic mice and* Cx3cr1* ^EGFP/+^ mice have shown a constant turnover of resident retinal microglia by replenishment from bone marrow-derived myeloid cells. They have also found that bone marrow-derived monocyte precursor cells are able to completely replace the retinal myeloid cell population within 6 months via migration over the blood-retinal barrier [16–19]. However, other authors have proposed that bone marrow-derived precursors only play a minor role in uninjured retina microglia maintenance and discovered that engraftment of bone marrow-derived microglia in the retina takes place almost only with retinal damage [17, 20].

The middle layer of the wall of the eye, the uvea, also presents networks of macrophages and DCs that participate in maintaining ocular homeostasis [21, 22]. Moreover, corneal immune privilege has been partly attributed to the lack of functional antigen-presenting cells (APCs) within the cornea [23].

Lymphoid cells are distributed as diffuse subpopulations all over the ocular surface [24]. It has been detected that DCs reside in the corneal epithelium [25], monocyte-lineage cells in the corneal stroma [26], and macrophages in the lamina propria of the conjunctiva [27]. Macrophage-like cells in the substantia propria of the conjunctiva perform a relevant phagocytic function on the outer ocular surface part, DCs in the corneal epithelium quickly respond to foreign antigens, and monocyte-lineage cells in the corneal stroma work as DC and macrophage precursors and also possess phagocytic activity.

The lamina propria is rich in bone marrow-derived cells that form a mucosal immune system of different kinds of blood vessels. This system is known as the conjunctiva-associated lymphoid tissue (CALT). Generally, mucosa-associated lymphoid tissue (MALT) represents a part of the immune system located on mucosal surfaces and consists of clusters (follicles) of lymphatic cells situated within and beneath the epithelium of a mucosal surface. The cellular components of MALT include B-cells, CD4^+^ and CD8^+^ T-cells, antigen-presenting dendritic cells, macrophages, and, occasionally, mast cells and eosinophils in the interfollicular region [28]. In the eye, its most well-known representatives are conjunctiva-associated lymphoid tissue (CALT) [29] and lacrimal duct-associated lymphoid tissue (LDALT) [30].

Like resident phagocytic cells, macrophages are armed with a wide array of surface receptors that are specific for pathogens or antigens, which render them efficient to play their role in steady-state tissue homeostasis: clearance of apoptotic cells, phagocytosis of foreign material, and production/secretion of growth factors and proinflammatory cytokines. Microglial cells also present additional roles in retinal growth by phagocytosing the pyknotic cells generated in the developing retina and by contributing in retinal neurogenesis and blood vessel formation [31–34].

In both phenotype and function terms, macrophages have enormous heterogeneity, which shows the specialization of tissue-resident macrophages in microenvironments. The M1/M2 macrophage activation paradigm has provided a useful framework, but a more comprehensive classification is evidently required. So the M2 label includes cells with marked differences in their biochemistry and physiology. In fact, it seems that macrophages do not form stable subsets. Rather than subsets of macrophages, we find pathways that interrelate to form complex, and even mixed, phenotypes [35–37].

Classical DCs are APCs with the unique ability to induce primary immune responses, armed with strong phagocytic activity as immature cells and good cytokine-producing capacity as mature cells. DCs detect and transfer foreign information to the cells of the adaptive immune system. Hence, DCs not only are crucial for inducing primary immune responses, but also may be relevant for generating immunological tolerance and for the regulation of the T-cell-mediated immune response type [18, 38]. Thus, during human uveitis, the aqueous humor suppression of DCs helps maintain immune regulation in the eye [39].

## 3. Blood-Ocular Barriers and the Eye's Immune Privilege

Delicate ocular structures cannot tolerate intense inflammation without loss of integrity and vision. Accordingly, the eye has its own mechanism to protect itself from inflammation called immune privilege. Immune privilege is meant to be an adaptation to restrict immune-mediated inflammatory processes that might inflict injury to eye cells with limited regeneration capacity [40]. Basically, it consists in active tolerance to foreign antigens through different mechanisms that may lastly induce apoptosis, promote the production of anti-inflammatory cytokines, and mediate the activation of antigen-specific regulatory immunity. These mechanisms also attempt to impose themselves upon immunity within the uveitic eye [41].

Multiple anatomical, structural, physiological, and immunoregulatory processes converge to maintain this so-called immune privilege. First, the carefully sealed blood-ocular barriers (the blood-aqueous barrier and the blood-retinal barrier) limit the passage of inflammatory cells and molecules from blood into the eye. Second, other mechanisms promote the eye's immune privilege, such as lack of lymphatics, intraocular immune modulators, induction of T regulatory cells (Tregs), and other properties that maintain tissue integrity [42]. So while some periocular tissues contain lymphatics (e.g., conjunctiva and the episclera), the eye's intraocular compartment lacks traditional lymphatics. Immune effectors in the blood stream, including sensitized T-cells and antibodies, are mostly excluded from the eye by potent blood-ocular barriers [43].

The extraocular counterpart of intraocular immune privilege is ocular surface mucosal tolerance. The conjunctiva consists of an epithelium and an underlying free connective tissue named the lamina propria. Epithelial histology is stratified nonsquamous and comprises two to three cell coats that display a typically cuboidal morphology [24]. As a mucosa, it is ensured by the mucosal immune system (innate and adaptive) present in the tissue and tear film. The immune system of the ocular surface forms an eye-associated lymphoid tissue (EALT). Mucosa and therefore EALT have certain characteristics that differentiate them from the central immune system and result in mechanisms such as immunological ignorance, tolerance, or an immunosuppressive local microenvironment, all of which favor nonreactivity and anti-inflammatory immunological responses. The interaction of all these mechanisms also results in the immune privilege of the ocular surface [24].

The inner blood-retinal barrier is set within the retinal parenchyma and consists of a nonfenestrated endothelium that is interconnected by tight junctions and covered by foot branches of astrocytes. The inner blood-retinal barrier may display a proinflammatory environment upon pathological induction. Nevertheless, the outer blood-retinal barrier facilitates intraocular migration by leukocytes and is equipped with immunoregulatory skewing capacities [44]. This barrier is set exteriorly to the neural retina and consists of tight junction-interconnected retinal-pigmented epithelial cells and fenestrated choriocapillaris. Finally, the blood-aqueous barrier is formed by the nonpigmented ciliary epithelium of the ciliary body and the vascular fenestrated endothelium of iris vessels. This barrier is permeable for cellular migration and leukocytes may access aqueous humor through its structure [44].

The placement of a foreign antigen into the eye elicits the generation of peripheral tolerance, which is maintained by antigen-specific Tregs. Specifically, the placement of alloantigens into the anterior chamber induces a form of immune tolerance known as anterior chamber-associated immune deviation (ACAID), which induces antigen-specific CD8^+^ Tregs and contributes to the eye's immune privilege by downregulating immune responses [41]. Treg cells prevent autoimmune diseases by maintaining self-tolerance and suppress pathogen-induced immunopathology.

Along with the induction of Treg cells, we find the induction of noncomplement-fixing antibodies [45]. Hence, systemic, antigen-specific, active immunologic tolerance is mediated by specific class I major histocompatibility complex- (MHC I-) restricted Tregs, which modulate inflammatory responses within the eye and form part of the immunoregulatory process. ACAID is a crucial mechanism for maintaining the eye's immune privilege. In an initial ocular phase, a foreign antigen placed in the anterior chamber is captured by indigenous APCs. In the eye, professional APCs, such as DCs, macrophages, and B-cells, form an integral part of the immune system. Today, we know that the process is mediated by macrophages (F4/80^+^ class II^+^ cells) that work as professional APCs, lie in the eye's anterior chamber, and are responsible for transporting the injected antigen to the spleen. In the spleen, eye-derived CD1-APCs come to rest in the marginal zone and secrete transforming growth factor-*β* (TGF-*β*) and macrophage inflammatory protein 2 (MIP2) by chemoattracting natural killer T- (NKT-) cells to the site. So ocular APCs interact with invariant B-cells and natural killer T-cells to generate multicellular clusters, which create immunomodulatory microenvironments in the splenic marginal zone which are rich in active TGF-*β*, interleukin 10 (IL10), and chemokine (C-C motif) ligand 5 (CCL5 or RANTES). The process culminates in the elicitation of systemic regulatory immunity through the induction of Treg cells [41, 46–48]. Thus, the immune response to antigens' contact results in an immunosuppressive environment, which includes the generation of Treg cells by aqueous humor, IFN-*γ* production, and inhibition of T-cell proliferation. Throughout this process, TGF-*β* has been identified as a critical factor. In fact, the macrophages treated with TGF-*β*2 and antigen are highly tolerogenic* in vivo* and induce antigen-specific and long-lasting tolerance in mice via the induction of Treg cells [49]. It is noteworthy that suppressor immunity in the eye is, therefore, expressed by the induction of Treg cells which, as regulators, suppress the induction and expression of T-helper cell type 1 (Th1) and Th2 immune expression systems [41].

Other mechanisms that support the eye's immune privilege include absence of MHC class II^+^ professional antigen-presenting cells [50] and the presence of immune modulators, such as TGF-*β*, complement decay accelerating factor (CD55 or DAF), cluster of differentiation 200 (CD200), cluster of differentiation 46 (CD46), macrophage migratory inhibition factor (MIF), and PD-L1 [41, 51].

Recently, Hsu et al. [52] showed that ACAID induced* in vivo* by the intravenous inoculation of* ex vivo* generated retinal antigen-pulsed tolerogenic antigen-presenting cells (TolAPC) in the presence of experimental autoimmune uveitis-inciting antigen (interphotoreceptor retinoid-binding protein, IRBP) or retinal antigen extract was able to modulate the clinical symptoms and inflammatory cytokines of IRBP-induced experimental autoimmune uveitis in mice. These results raise the possibility that the clinical symptoms of human uveitis might be relieved by a therapy that uses a target tissue extract instead of a specific antigen.

In any case, the immune privilege is a complex dynamic phenomenon that is the total sum of the processes and molecules which prevent the induction and expression of both innate and adaptive immunity. However, immune privilege incurs a significant risk; if the eye's privileged status is compromised, the succeeding disease can prove devastating.

## 4. Macrophages in Uveitis

### 4.1. Phagocyte Response to Infection

From sentinel and clearance functions, resident macrophages in tissues may induce acute inflammatory and vascular changes through their close association with the microvasculature. Acute inflammation is a process initiated by those cells that were previously present in affected tissues, mainly resident macrophages and dendritic cells. These cells are able to recognize the molecules that are linked to groups of the pathogens, pathogen-associated molecular patterns (PAMPs), or damage-associated molecular patterns (DAMPs) released from injured cells through the receptors contained on their surface or within these cells, namely, pattern recognition receptors (PRRs), for example, phagocytic C-type lectin receptors, scavenger receptors, nucleotide-binding oligomerization domain-like receptors (NLRs), and transmembrane signaling Toll-like receptors (TLRs) [53, 54]. When PRRs bind PAMPs, they trigger an immediate cellular or molecular response.

Firstly, macrophages engulf and kill invading microorganisms and present an important phagocytic role as first defense in innate immunity but also have pathogens and infected cells that are targeted by an adaptive immune response. Macrophages release different toxic products that help phagocyte microorganisms, which include antimicrobial peptides, reactive nitrogen species (NO), and reactive oxygen species (ROS), such as superoxide anion (O_2_^−^) and hydrogen peroxide (H_2_O_2_) [55–57]. In the early phase of the uveoretinitis, concomitantly with the generation of oxygen metabolites, peroxidation of retinal membrane lipids takes place [58, 59].

Macrophages also support initial inflammation by secreting signaling proteins (cytokines and chemokines) that activate other immune system cells and recruit them in an immune response [60]. Thus, some NLRs also recognize nonmicrobial danger signals and form large cytoplasmic complexes called inflammasomes, which link the sensing of microbial products and metabolic stress to the proteolytic activation of proinflammatory cytokines IL-1*β* and IL-18 [61]. It is also known that ROS production activates the inflammasome [62].

Phagocytic myeloid cells, particularly DCs, also utilize phagocytosis to direct antigens to both compartments MHC I and MHC II [23, 63]. Hence, phagocytosis plays a dual role as an effector of innate immunity and an inductor of acquired immunity.

### 4.2. Macrophage Activation

Nonactivated inflammatory monocytes and inflammatory DCs work like APCs, but they suppress T-cells through nitric oxide production when activated [64]. Suppressive inflammatory monocytes are induced by IFN-*γ*, GM-CSF, TNF-*α*, and CD154, which derive from activated T-cells during their interaction. Zhu et al. [65] proved macrophage plasticity in experimental autoimmune encephalomyelitis. These authors demonstrated that the myeloid cells isolated from the CNS in different disease stages were able to either activate or suppress T-cells.

The term macrophage activation (classical activation) was introduced by Mackaness in the 1960s in an infection environment [66], was then linked with Th1 responses and IFN*γ* production [67], and was later extended to cytotoxic and antitumoral properties [68]. Macrophages respond to diverse environmental signals by expressing an array of functional phenotypes [35]. Therefore, with different pathological stimuli, for example, injury or damage, microbial attack, or activated lymphocytes, macrophages undergo reprogramming, which gives rise to diverse macrophage phenotypes, broadly classified as the M1 proinflammatory (classically activated) phenotype and an M2 anti-inflammatory (alternatively activated) one, which are two extremes of a range of macrophage functional states [35–37].

Polarized macrophages vary in terms of receptor expression, effector function, and cytokine and chemokine production. Thus, when mirroring the Th1-Th2 paradigm, they are considered to be M1 macrophages when induced by microbial products or proinflammatory cytokines, such as IFN*γ*, lipopolysaccharide, TNF, GM-CSF, or Toll-like receptor ligands, and M2 anti-inflammatory macrophages when stimulated by Th2 cytokines like IL4, IL10, IL13, IL21, IL33, or TGF-*β* [66, 69–72].

### 4.3. Macrophages in Autoimmune-Induced Uveitis (AIU)

The most widely used uveitis animal model is experimental autoimmune uveoretinitis (EAU). EAU is an animal model of human endogenous uveitis that is normally induced by several retinal autoantigens [73]. Thus, the pathology of EAU accurately resembles human uveitic diseases of a recognized autoimmune nature in which patients display immunological responses to retinal antigens (sympathetic ophthalmia, Behcet's disease, and birdshot retinochoroidopathy, among others) [51, 74]. Traditionally, the EAU mice model has been induced with retinol-binding protein- (RBP-) 3, previously known as interphotoreceptor retinoid-binding protein, a major retinal protein that shuffles vitamin A derivatives between photoreceptor cells and the retinal pigment epithelium. RBP-3 is emulsified in complete Freund's adjuvant, which consists in a suspension of tuberculosis bacteria in mineral oil, which is crucial for disease induction in both mice and rats [74]. Another induced EAU model is the “humanized” model of EAU, which is induced in HLA class II transgenic mice. Thus, modified transgenic mice for any one of a number of HLA class II alleles develop EAU after immunization with retinal arrestin [75, 76]. Different mouse models of spontaneous disease have also been achieved such as in RBP-3 T-cell receptor transgenic (R161H) mice [77], also in mice deficient in the AIRE (autoimmune regulator) gene [78], and in HLAA29 transgenic mice [79] or by neoantigens (hen egg lysozyme, HEL) [80].

Clinical disease in EAU depends on both CD4^+^ T-cells and macrophages. Hence, numerous studies have identified macrophages that play a crucial role in EAU, mostly in relation to early stages: during the induction and effector phase of the disease [81–83]. So certain evidence shows distinct roles for macrophages in different stages during EAU evolution and in relation to M1 classical activated macrophages, promoted in a TNF-dependent manner by the IFN gamma release from the Th1 CD4^+^ T-cell infiltrate [84]. TNF-mediated macrophage activation is critical for the clinical manifestation of uveitis. In fact, Khera et al. [85] showed that a soluble form of TNF is required in EAU for inflammatory cell infiltration into the target tissue. However, at the tissue site, inhibition of both soluble TNF and transmembrane TNF is required to inhibit macrophage activation and to protect from tissue damage. In this context, tissue damage is mediated by the further release of proinflammatory cytokines (IL1b, IL6, and TNF*α*), ROS, and nitric oxide [84, 86]. Hence, IFN*γ*-mediated macrophage activation, which depends on TNF-*α* and functional TNFR1, results in high levels of nitric oxide, TNF-*α*, and IL-6 and thus produces lipid peroxidation and damages surrounding cells [87, 88]. In a recent study, London et al. [89] showed that CCR2^+^CX_3_CR1^low^Ly6C^+^ monocyte-derived macrophages are recruited early during the disease process and are involved in the induction of EAU pathology by displaying the typical proinflammatory phenotype. Early inhibition of this infiltration has been seen to prevent EAU, whereas its inhibition after the disease peaked resulted in fewer Foxp3^+^ Treg cells in the retina and also in worse disease outcome.

Some studies have also demonstrated the presence of macrophages in the resolution phase, with infiltration of myeloid cells showing suppressive properties to T-cell activation and proliferation which, in a sense, regulates the autoimmune response through both nitric oxide activation-induced cell death and the elimination of T-cells in the retina [86] through prostaglandin-mediated pathways [88]. Activated macrophages release TNF*α*, and the expression of one of its receptors, TNFR1, is necessary for normal organ-specific autoimmunity development and is a critical key for developing the macrophages that control T-cell proliferation. Thus, TNFR1^−/−^ macrophages are unable to suppress T-cell proliferation. Lack of TNFR1 is also associated with lack of PGE2 production [88]. PGE2 is required for myeloid-derived suppressed cells maturation* in vivo* and can modulate the function of dendritic cells, such as APCs [90, 91].

Some recent reports in the experimental autoimmune encephalomyelitis model have revealed that invariant NKT cell activation results in the differentiation of monocytes in an M2 phenotype and has a positive impact on disease development [92]. Similarly, London et al. [89] found a subset of macrophages (CX_3_CR1^high^) with immune-resolving activity in EAU that enabled the disease to reach a state of equilibrium instead of relapsing. These authors also detected an anti-inflammatory mediator, IL10, expressed by the infiltrating macrophage. IL10 has been shown to play a protective role in EAU [93] and is an important factor in the development and function of Tregs [94, 95].

### 4.4. Macrophages in Endotoxin-Induced Uveitis (EIU)

Acute anterior uveitis (AAU) is the commonest form of uveitis as it represents up to 92% of all uveitis cases [96, 97]. AAU is characterized by the breakdown of the blood-aqueous barrier and acute inflammation of the iris and ciliary body, by showing the upregulation of cell adhesion molecules on the uveal vasculature, as well as the aqueous humor expression of proinflammatory cytokines (e.g., TNF*α* and IFN*γ*) and chemokines that selectively recruit and activate inflammatory cells (neutrophils, monocytes, and lymphocytes) into the uvea and anterior chamber. Since Rosenbaum et al. [98] described how systemic immunization with endotoxin (lipopolysaccharides) from Gram-negative bacteria produces bilateral acute anterior uveitis in rats in 1980, many clinical and experimental data have shown that any kind of Gram-negative bacteria or their lipopolysaccharides (LPS) play a key role in AAU development [97]. Indeed, clinical and laboratory research has verified that Gram-negative bacteria species, such as* Klebsiella*,* Salmonella*,* Yersinia*, and* Shigella*, can trigger AAU [99].

Hence, endotoxin-induced uveitis (EIU) is an acute form of uveitis that is induced by injecting a sublethal dose of LPS, a component of the cell walls of Gram-negative bacteria, into experimental animals, including rats, mice, and rabbits. Therefore, it is an advantageous human AAU model that is not autoimmune that has acted as a valuable model for ocular acute inflammatory processes driven by innate immune mechanisms and their effects on tissue. EIU is marked by both vasodilatation of the iris and vascular changes in the ciliary body, accompanied by increased vascular permeability and a breakdown of the blood-aqueous barrier [100]. In the eye's anterior segment, it involves the activation of endothelial cells and resident monocytes-macrophages as initiators of inflammatory response, following neutrophil and mononuclear cells infiltration, to generate multiple proinflammatory mediators, such as cellular adhesion molecules, cytokines and chemokines, ROS production, and tissue damage [100, 101]. Inflammation ensues 4 h after LPS injection, peaks after 24–48 h, and declines 96 h after disease induction.

Monocytes begin to marginate in iris vessels at 2 h after injection; after 4 h, they form perivascular cuffs and are broadly distributed among resident macrophages after almost 24 h [102]. Therefore, monocytes and macrophages respond directly to LPS as an initial source of cytokines and chemokines.

EIU is usually considered an inflammation of the anterior uvea. However, posterior segment findings have also been reported [103], including vitritis, vitreous hemorrhage, retinal vasculitis, inflammatory cell infiltration of the retina, and choroiditis. Thus, systemic LPS injection has been followed by the massive entry of macrophages into the retina, although major histocompatibility class II-positive cells have not been found after LPS injection [104]. Therefore, EIU alters the expressions of Kir4.1 and AQP4 in the retina, which indicates a disturbance of water and potassium transport in the retina that contributes to retinal edema during ocular inflammation [105]. Choroid is also severely inflamed after systemic LPS administration, with a massive influx of ED1-positive macrophages into the area below the retinal pigment epithelium [106].

Intraocular macrophages play a key role in EIU. TLRs are a family of phylogenetically conserved PRRs of the innate immune system that recognize unique PAMPs. Indeed, a marked TLR4 protein expression pattern has been observed in the human uvea [107]. TLR4 is expressed in macrophages as the main specific LPS recognition [108] and cellular activation signaling receptor, which may play a relevant role in starting uveitis.

TLR4 is unique among TLRs as it signals through two well-characterized adaptor molecules: MyD88 and TRIF [109]. However, EIU is primarily dependent on both radiation-resistant cells and MyD88, but not on TRIF [110]. The TLR4 signaling pathway induces NF*κ*B activation and, consequently, the synthesis and release of proinflammatory mediators such as TNF-*α*, cytokines, chemokines, adhesion molecules, ROS, and reactive nitrogen radicals [111]. Chen et al. [112] observed a preferential expression of TLR4 in tissue macrophages within the iris and ciliary body in EIU and proposed a novel mechanism for the initiating factors and immunopathogenesis of uveitis. In fact, inducing acute anterior uveitis in TLR4 gene-deficient mice in an EIU model was not possible [113]. Yang et al. [114] showed that TLR4 signal activation can lead to the cascading expansion effect of cytokines during EIU by analyzing cytokine changes in the supernatant of cultured macrophages in diverse mice groups after LPS stimulation. LPS also functions as an antagonist of peroxisome proliferator-activated receptor alpha (PPAR*α*), a nuclear transcription factor with protective effects associated with the modulation of TLR4 signaling pathways in EIU [115]. A recent study by Ekici et al. [116] has shown that a TLR4 antagonist treatment in the EIU rat model significantly lowered the levels of TNF*α*, MDA, and NF*κ*B. The global result was less inflammatory damage in terms of serum and retinochoroidal tissue parameters.

Finally, in recent studies conducted into EIU, M1 and M2 macrophage polarization has been reported [117, 118].* In vitro*, LPS induces a typical M1 profile through the recognition of LPS by TLR4 [119, 120]. Signaling mechanisms include NF-*κ*B activation, LPS-induced TNF-*α* factor upregulation, and PI3K pathway stimulation [121]. During an inflammation resolution process of EIU mediated by lipid-derived protein Resolvin D1 (RvD1), Rossi et al. [117] found that LPS- and RvD1-treated rats reduced the presence of M1 macrophages and increased protective M2 macrophages in ocular tissues.

## 5. Conclusions

Resident and infiltrated macrophages play relevant roles in uveitis as effectors of innate immunity and inductors of acquired immunity. In the last few years, experimental animal models have enabled us to better understand the leading role of macrophages in eye inflammation processes, counting macrophage polarization and the significant role of TLR4. This knowledge should guide advantageous iterative research, including strategic* in vitro* and* in vivo* studies, to establish mechanisms and possible therapeutic targets for human uveitis resolution.
